# Treatment of open fractures with a computer-assisted external fixator system without the use of fluoroscopy

**DOI:** 10.1186/s13018-016-0379-9

**Published:** 2016-04-26

**Authors:** Adnan Kara, Haluk Celik, Ali Seker, Ozgur Karakoyun, Raffi Armagan, Ersin Kuyucu, Mehmet Erdil

**Affiliations:** Department of Orthopaedics and Traumatology, Faculty of Medicine, Istanbul Medipol University, Istanbul, Turkey; Department of Orthopaedics and Traumatology, Zonguldak Ataturk State Hospital, Tepebası Mah, Kapuz Cad. Turkuaz apt. No: 28/10, 67030 Zonguldak, Turkey; Faculty of Medicine, Department of Orthopaedics and Traumatology, Namık Kemal University, Tekirdag, Turkey; Department of Orthopaedics and Traumatology, Sisli Hamidiye Etfal Research Hospital, Istanbul, Turkey

**Keywords:** Open fracture treatment, Circular external fixators, Fluoroscopy, Computer-assisted circular external fixators

## Abstract

**Background:**

Developed for the treatment of deformity correction, computer-assisted circular external fixators in recent years have also been used for fracture fixation. In this study, we aimed to present the efficacy of computer-assisted circular external fixator use in open long bone fractures with our new technique.

**Methods:**

Between February 2013 and June 2014, 14 patients (mean age 24.5 (range 20–32)) with open tibial or femoral open fractures were treated with the computer-assisted fixation system (Spider Frame-Tasarım Medikal, Istanbul, Turkey). In all patients, appropriate positions of the rings and Schanz screws were determined by measurements on preoperative radiographs. The length of the Schanz screws were determined by depth measure marks on drill bits. Obvious deformities were corrected intraoperatively by manipulations, but residual deformities were corrected by a software program (Spiderfix, Tasarım Medikal, Istanbul, Turkey). We did not use fluoroscopy during the procedures.

**Results:**

Ten patients had tibia diaphyseal and four patients had femoral diaphyseal fractures. Mean surgical time was 24.2 (range 18–28) min. Average follow-up time was 10.2 (range 9–14) months. Mean time for deformity correction was 3.1 (2–5) days. Complete union was observed in all patients with a mean of 4.9 (range 3–9) months. There were two grade 2 pin site infections treated with oral antibiotherapy and pin site care. We did not detect any Schanz screw breakage, loosening, deep infection, nonunion, or malunion.

**Conclusions:**

Computer-assisted external fixation systems can be used in the treatment of open fractures, and they provide the chance for acute or gradual correction. Preoperative planning and assistant devices with depth measures may decrease the procedure time and the need for fluoroscopy use.

## Background

Open fractures can be difficult to manage and may cause complications such as infection, nonunion, and neurovascular injuries [1]. It is difficult to decide on the use of internal fixators, particularly for those patients presenting late and who do not get an initial treatment.

Ilizarov presented his circular fixator concept, and many surgeons use these systems in the treatment of long bone open fractures [2, 3]. A long learning curve, difficulties during applications, and the need for fluoroscopy during procedures are the main disadvantages [4, 5]. It is possible to overcome these disadvantages with computer-assisted circular fixator systems [6–8].

Preoperative planning of the Schanz screw configuration and the use of drills with a length indicator enable easy surgery and short operative times without the need to use the C-arm fluoroscope.

A Stewart platform is used in several hexapod circular external fixation systems. We used such a system (Spider Frame-Tasarım Medikal, Istanbul, Turkey), which has two frames and six rods. The system has the ability to correct multiplanar deformities by the help of its software system and can also be used in the treatment of nonunions and fractures. We used this system in the treatment of open tibial and femoral fractures without the help of the fluoroscopy, and we aimed to present the advantages of this technique.

## Methods

**Fig. 1:**
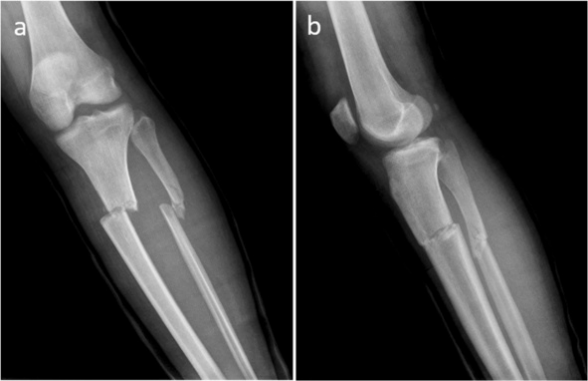
**a**, **b** Twenty-eight-year-old male with a type 2 open tibia fracture. The patient was referred to us after 1 week. Because of infected wound debridement and osteosynthesis with Spider Frame was made

Between February 2013 and June 2014, a total of 14 male patients was operated on for open lower extremity long bone fractures and treated by using the Spider Frame. All patients were domestic war victims injured in another country and were transferred to our clinic without initial debridement and treatment. Gustilo-Anderson [9] type 2 and 3A open fractures were included (Fig. 1). All patients were operated on by two experienced trauma surgeons. The study was approved by the Ethical Committee of Namık Kemal University, Faculty of Medicine (reference number: 2015/127/11/10). Consent to participate was obtained from all participants, and consent to publish was obtained for all images and patient details presented in this study.

### Surgical technique

The appropriate localizations of the frames and Schanz screws were determined on the anteroposterior and lateral radiographs preoperatively. We measured the distances between the fracture and the adjacent joints (knee and hip joints for femoral fractures, ankle and knee joints for tibial fractures). Under spinal anesthesia, patients were operated on in the supine position. One milligram of Cefazolin sodium was used for prophylaxis 30 min before the procedure. After debridement and irrigation of the wound, reference points for the proximal ring were drawn after the measurement of the distance with a ruler from the knee joint line according to the preoperative calculations. A Kirschner wire was inserted perpendicular to the long axis of the bone and fixed to the reference ring. Six telescopic struts were adapted to the rings, and two or three Schanz screws were inserted for each ring. The wounds were closed primarily in all patients.

**Fig. 2:**
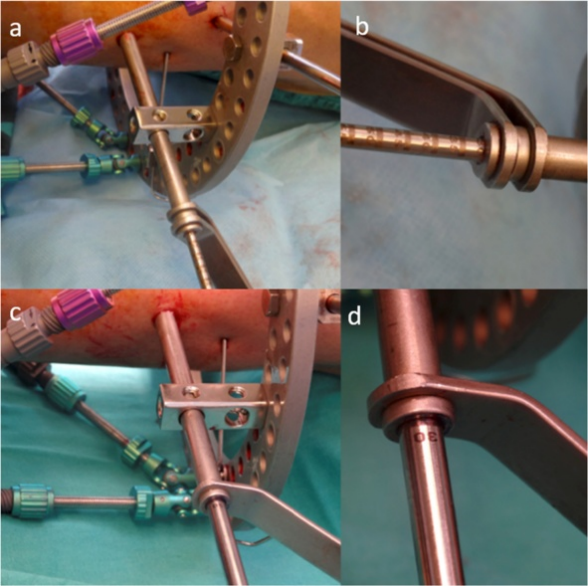
Schanz sleeve (**a**), drill sleeve (**b**), drill bit (**c**), and Schanz screws (**d**) with depth measure marks

Schanz sleeves, drill sleeves, drills with depth marks, and Schanz screws were used in this technique (Fig. 2). Entry points, which had been decided preoperatively, were marked during the procedure. A 0.5-cm stab incision was done, and a large Schanz sleeve, which contains a drill sleeve inside it, was advanced up to the bone. During drilling, we stopped advancement just after drilling of the far cortex, and we measured the bicortical distance from the depth measure marks on the drill bit. Schanz screws with measure marks were advanced according to the measured distances. Unmarked hydroxyapatite-coated Schanz screws were marked with a marker and advanced up to this mark. We did not use fluoroscopy during the insertion of the Schanz screws.

### Postoperative management

**Fig. 3:**
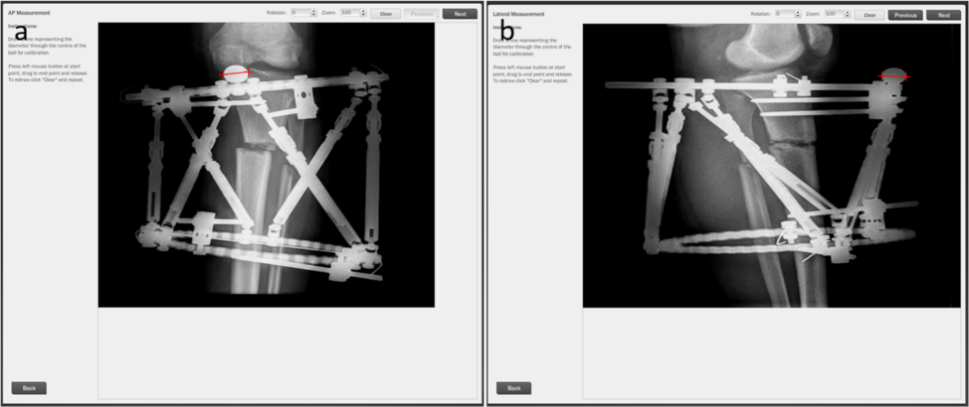
**a**, **b** Correction of deformity with Spiderfix software

**Fig. 4:**
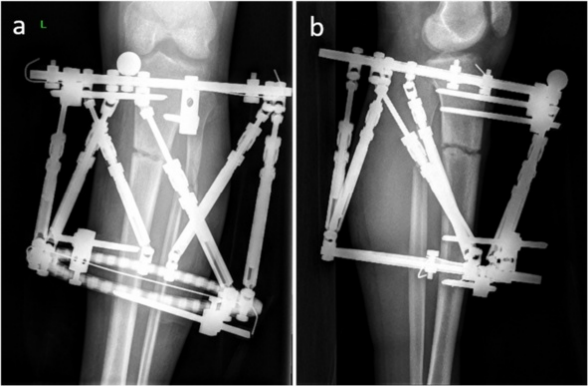
**a**, **b** The postoperative third-day radiograph of the fracture after correction of the deformity

**Fig. 5:**
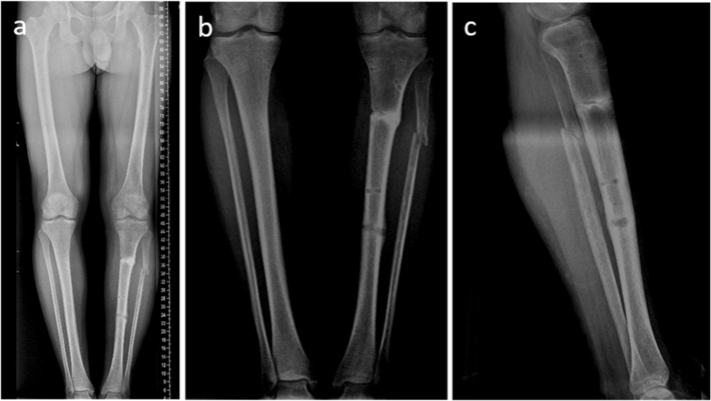
**a**–**c** The postoperative fifth-month follow-up radiographs of the patient showed complete union of the fracture

**Fig. 6:**
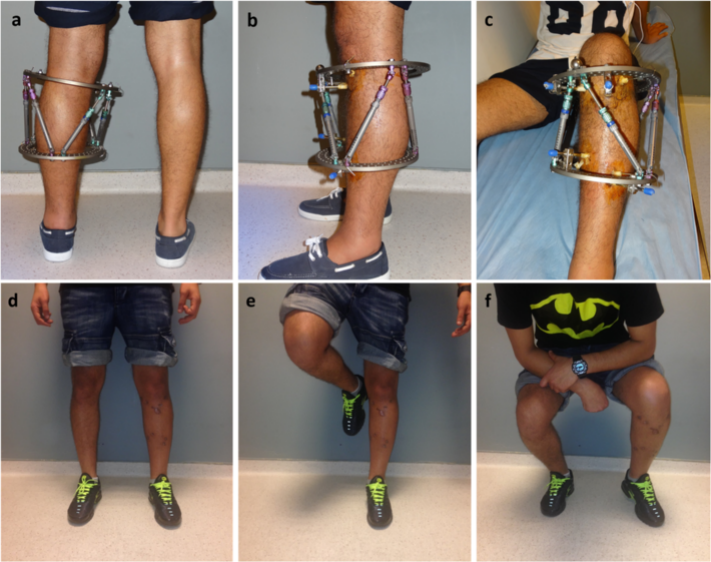
The postoperative fourth-month follow-up clinical photos of the patient (**a**–**c**) and fifth-month follow-up clinical photos of the patient (**d**–**f**)

Anteroposterior and lateral radiographs were obtained postoperatively. The reference ring has to be straight in those radiographs. Deformities were corrected starting from the postoperative first day by using a software program (Spiderfix; Artificial Neural Network, Tasarım Medikal, Istanbul, Turkey) (Fig. 3). After correction of the deformities, the systems were locked and weight bearing was allowed with two crutches as tolerated (Fig. 4). Patients were followed with an interval of 45 days. Fixators were removed after observation of the complete union of at least three cortices. (Figs. 5 and 6) We investigated the surgical time and complete union time, any pin site infection, deep infection, implant failure, malunion, or nonunion.

## Results

Mean age of the patients was 24.5 (range 20–32) years at the time of surgery. Mean follow-up was 15.5 (12–23) months. All patients were transferred from another country. The causes of fractures were gunshot injury in 12 patients and fall from height in two patients. Mean presentation time following injury was 5.6 days (4–8 days). Lack of initial treatment and the late presentation led us to not perform initial treatment. Ten patients had tibial fractures, and four patients had femoral fractures. Of these, two had 42 C3, three had 42 B2, three had 42 B3, two had 42 A3, two had 32 C1, one had 32 B1, and one had 32 B2 fracture according to the AO classification system. Concomitant injuries were discovered in six patients, as follows: three forearm, two metatarsal, and one calcaneal fractures. Twelve fractures were type 3A, and two fractures were type 2 open fractures. Mean procedure time was 24.2 (18–28) min. Mean time for deformity correction was 3.1 (2–5) days.

### Outcome evaluation

There were two grade 2 pin site infections according to Paley [10]. These patients were treated with oral antibiotherapy and pin site care. At the last follow-up control, 10 patients had experienced complete union. Four patients with femoral fractures had residual deformity less than 5°. No neurovascular injury or Schanz screw loosening/fracture was observed. Average union time was 4.9 (range 3–9) months. Following the fracture healing, fixators were dynamized and removed 2 months after that.

## Discussion

Urgent debridement, antibiotherapy, and stable fixation were the principles of the treatment of the open fractures [11]. Patients in this study had open fractures of long bones and did not have an initial treatment. Since they presented a mean 5.6 days after injury, we believed that external fixation would be more beneficial than internal fixation. With our technique and preoperative planning, we had a short operative time (mean 24.2 min.). Moreover, we did not use C-arm fluoroscope. All patients had fracture healing without any complications.

Having a gradually increasing use, computer-assisted external fixators combine the basic Ilizarov principles with computer software and come in many types. Those fixators that have similar working principles include having two circular Ilizarov frame and six telescopic rods, which have a universal hinge on either end. Length indicators on these rods enable changing of the length. A Spider Frame requires 13 parameters in order to correct the deformity or reduce fractures. Of these parameters, six belong to skeletal deformity, three belong to frame elements, and four belong to the position of the frame according to the extremity. These parameters are transferred to computer software. The final correction schedule enables correcting the deformity just by changing the lengths of the rods. Both deformity and mounting parameters are calculated according to origin. In the planning of reduction with a Spider Frame, the surgeon defines the reference fragment and frame. The reference point is important in calculating the mounting parameters according to the origin. Translation in mounting parameters is calculated according to the center of the reference frame.

There are different options for fixation. Plate-screw fixation is mainly used in metaphyseal fractures, but it is not recommended to use this system in type 3 open fractures due to the infection risk [2]. Reamless intramedullary nails and external fixators can also be used [12]. There is no significant difference between these two treatment methods in fracture healing time, deep tissue infection, and osteomyelitis in open tibial and femoral fractures [13]. Traditional circular fixators need excellent preoperative planning and careful application [5]. Computer-assisted external fixation systems are based on Ilizarov principles and provide similar stability to that of Ilizarov-type external fixators with easier application. Nowadays, they are more commonly used in the treatment of open fractures [5, 14, 15]. This study presents the use of the Spider Frame in open fractures, and to our knowledge, is the first paper in the literature about this computer-assisted system.

Fluoroscopy is widely used during procedures, especially in minimally invasive surgeries, but it also causes exposure to ionizing radiation during surgery. The use of fluoroscopy more than 1.7 min causes an increase in radiation exposure [16]. It has been shown that orthopedic surgeons have more risk of tumors in a study with 24-years follow-up [17]. In this study, we did not use fluoroscopy during the procedures.

Correction of deformities can be planned either on the fluoroscopic view intraoperatively or plain radiographs postoperatively. Gantsoudes et al. reported more accurate measurements with fluoroscopic view during surgery [18]. In another study, no difference was detected between the two techniques [19]. We preferred to correct deformities postoperatively. In this technique, there is no need to adapt rings to the fractured bone. This advantage makes the surgical technique easier and decreases the use of fluoroscopy.

We determined the Schanz screw insertion points preoperatively, and during surgery, possible entry points were determined after measuring the distance with a ruler from reference points such as the adjacent joint line. We used depth-numbered drill bits and determined the length of the Schanz screws. We tried to reduce the fracture manually during surgery and fixed the system.

Our technique could be applied in all long bone fractures. Elimination of the use of fluoroscopy, short surgery time, the correction of the deformity without the need for a second surgery, and ease of hardware removal are some advantages of the technique. High cost and the necessity to learn computer software are the disadvantages.

There are several limitations of this study. The number of patients was low, and the patients were evaluated retrospectively. In addition, those systems are more expensive than ordinary circular fixators. Our technique does not need fluoroscopic control during surgeries, but determination of the length of the Schanz screws was performed by measurement from drill bits. Therefore, experience and the sensations of the surgeon are important parameters; otherwise, the application of this technique can be problematic in osteoporotic patients.

## Conclusions

Computer-assisted external fixation systems can be used in the treatment of open shaft fractures. They provide the chance for acute or gradual correction. Preoperative planning and assistant devices with depth measures may decrease the procedure time and the need for fluoroscopy use.
